# Panton-Valentine Leukocidin and Staphyloccoccal Skin Infections in Schoolchildren^1^

**DOI:** 10.3201/eid1001.030144

**Published:** 2004-01

**Authors:** Karim Boubaker, Patrick Diebold, Dominique S. Blanc, François Vandenesch, Gérard Praz, Georges Dupuis, Nicolas Troillet

**Affiliations:** * Central Institute of the Valais Hospitals, Sion, Switzerland; † School Medicine Service, Monthey, Switzerland; ‡ Centre Hospitalier Universitaire Vaudois, Lausanne, Switzerland; § French National Reference Center for Staphylococcal Toxemia, Lyon, France

**Keywords:** *Staphylococcus aureus*, skin, furunculosis, toxins. Panton-Valentine leukocidin, Mupirocin, Chlorhexidine, handwashing

## Abstract

The Panton-Valentine leukocidin is associated with staphylococcal skin and pulmonary infections. We describe a school outbreak of skin infections and the public health response to it. Nasal carriage of a Panton-Valentine leukocidin–positive *Staphylococcus aureus* clone was detected only in previously ill classmates and their family members.

*Staphylococcus aureus* colonizes approximately 30% of the general population and up to 50% of persons who are intravenous drug users, diabetics, or healthcare workers. The organism is mainly transmitted between persons by close contact (*1*,*2*). In addition to its increasing ability to resist antimicrobial agents, *S. aureus* displays a wide array of virulence factors that render it capable of causing a larger spectrum of infections than any other bacteria (*1*).

Exotoxins constitute essential components of the virulence mechanisms of *S. aureus*. Nearly all strains secrete hemolysins, nucleases, proteases, lipases, hyaluronidase, and collagenase, which convert host tissues into nutrients required for bacterial growth (*3*). Some strains produce additional exoproteins that may be responsible for particular clinical manifestations, including the staphylococcal enterotoxins, the toxic-shock syndrome toxin-1, the exfoliative toxins, and the Panton-Valentine leukocidin (PVL) (*3*). PVL, a bicomponent cytotoxin encoded by two contiguous and cotranscribed genes carried on a bacteriophage, causes leukocyte destruction and tissue necrosis. This cytotoxin is produced by <5% of *S. aureus* isolates and has been associated with necrotic lesions involving the skin and with severe necrotizing pneumonia (*4*,*5*).

## The Study

**Figure 1:**
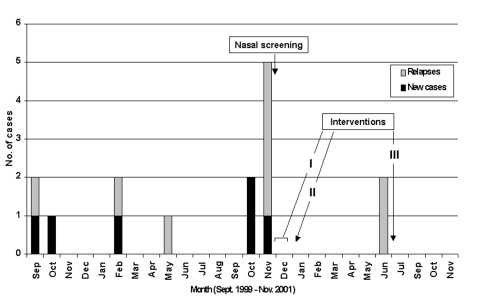
Cases of skin infections among schoolchildren, Switzerland, September 1999–November 2001. I: Nasal mupirocin twice a day, chlorhexidine showers once a day for carriers of penicillin-resistant *Staphylococcus aureus* and their family members (5 days); alcoholic hand rubs in the classroom and at home (3–4 weeks). II: Repeated measures (5 days) in those still found to be carriers and in their family members. III: Repeated measures limited to the two relapsing children and their family members.

 From September 1999 to November 2000, 6 of the 22 students from a single third-grade classroom in a town of 12,000 in western Switzerland had 13 episodes of skin infections, including furuncles, abscesses, and cellulitis. The outbreak peaked in October and November 2000, with three new cases and four relapses (Figure 1). Two children were hospitalized. Most patients were treated with systemic antimicrobial agents. Some needed surgical incisions and drainage. Six cultures were performed and consistently grew *S. aureus* resistant to penicillin and amoxicillin (PRSA) and susceptible to flucloxacillin, amoxicillin/clavulanic acid, cephalosporins, erythromycin, doxycyclin, clindamycin, trimethroprim/sulfamethoxazole, ciprofloxacin, and rifampin.

At the end of November 2000, after health authorities were notified, nasal screening for carriage of *S. aureus* was performed first in the 22 classmates and their 2 teachers and next in the families of those who had had skin infections or who had been found to be carriers of PRSA. Nasal cultures were collected by using rayon swabs moistened with 5% NaCl, rotated five times in both anterior nares. The samples were carried within 2 hours to the laboratory and kept overnight at 35°C in an enrichment Mueller-Hinton broth with 5% NaCl. They were then plated onto mannitol-salt agar and sheep blood agar. The plates were incubated at 35°C with 5% CO_2_ for 24 hours. Persons with mannitol-fermenting, coagulase-positive colonies were considered carriers of *S. aureus* after species were confirmed and antimicrobial susceptibility was determined in an automated system (Vitek 2, bioMérieux, France). PRSA were saved and subsequently typed by pulsed-field gel electrophoresis (PFGE) after digestion by *Sma*I. Three isolates representative of the three clones were forwarded to the French Reference Center for Staphylococcal Toxemia, where polymerase chain reaction (PCR)-based methods were used to detect genetic sequences encoding enterotoxins, exfoliative toxins, toxic-shock syndrome toxin-1, β-hemolysin, LukE-LukD leukotoxin, and PVL (*4*).

**Figure 2:**
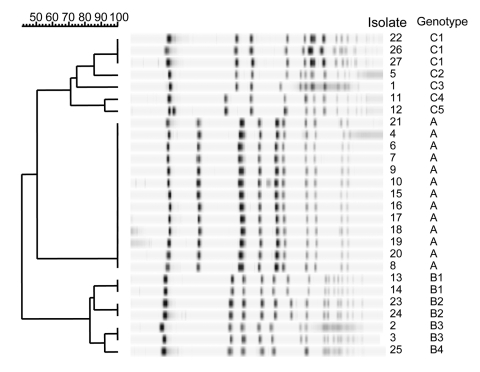
Band patterns of the *Staphylococcus aureus* isolates cultured from nasal swabs (pulsed-field gel electrophoresis). Some persons had two consecutive positive cultures (see text).

Ten of the 22 classmates and 1 of the 2 teachers (46%) were colonized with *S. aureus.* None harbored methicillin-resistant *S. aureus* (MRSA). Nine carried PRSA, including four of the six previously infected children (the other two had negative cultures). Two carried penicillin-susceptible isolates. The nine PRSA carriers and the two previously infected students with no nasal *S. aureus* had 36 family members, 3 of whom had recently also had skin infections. These three persons belonged to two families that included formerly infected classmates. Thirty-five family members were screened. Cultures grew *S. aureus* in 15 (43%), of whom 12 harbored PRSA and 3, penicillin-susceptible isolates. The results of PFGE, including PRSA isolates still found after the first set of preventive measures had been taken (see next paragraph), are shown in Figure 2. Genes encoding for PVL were found only in clone A (Table), which was harbored by three of the six previously infected classmates and six of their family members. The three clones showed the same susceptibility profile, which also corresponded to the profile found previously in the infecting isolates.

**Table:** Detection of genes encoding for various exotoxins, by clone^a^

	Clone A	Clone B	Clone C
β-hemolysin	–	–	–
Enterotoxins	G, I, K, L, M	G, I, K, L, M	H, M, O
Exfoliative toxins	–	–	–
LukD–LukE	+	–	–
Panton-Valentine leukocidin	+	–	–
Toxic-shock syndrome toxin-1	–	–	+

^a^Multiplex polymerase chain reaction, one isolate representative of each clone.

While waiting for PFGE and PCR results, we implemented measures derived from those in use for MRSA in hospitals (*6*). After information and teaching had been provided to the students and their parents, nasal mupirocin twice a day and chlorhexidine showers once a day for 5 days were prescribed for all the members of the nine families that included >1 PRSA carrier, regardless of clonal type. These measures were repeated a second time in five families because >1 of their members still harbored PRSA 1–2 weeks after the first set of measures was implemented. Moreover, alcohol-based hand rubs (500-mL multiuse containers) were used several times a day for 3 to 4 weeks in the nine families and by every student and teacher when entering and leaving the classroom.

One year later, no new case of skin infection had been detected among this population through active surveillance by the school nurse or periodic enquiries to the local pediatricians. Two children had relapsed 6 months later, in June 2001 (no culture available). Nasal mupirocin and chlorhexidine showers were repeated for 5 days in their families.

## Conclusions

 PVL has been associated with staphylococcal skin and pulmonary infections by means of retrospective and prospective studies comparing the clinical features of patients infected with PVL gene-positive or negative staphylococci (*4*,*5*). The intrafamilial spread of such strains (e.g., from mother to infant through breastfeeding) has been reported (*7*–*9*). To our knowledge, this report is the first on an outbreak attributable to a PVL-positive *S. aureus* clone that spread among schoolchildren and their families.

Lina et al. found that 50%-93% of *S. aureus* responsible for cutaneous abscess, cellulites, or furunculosis and 85% of those responsible for community-acquired pneumonia harbored the genes encoding for PVL compared with none of those causing diseases such as nosocomial pneumonia, infective endocarditis, urinary tract infection, enterocolitis, or toxic-shock syndrome (*4*). Gillet et al. found that 16 patients with community-acquired pneumona attributable to PVL-positive *S. aureus* were younger (median age 14.8 years), had less underlying disorders, and had more often had influenza-like syndromes or furuncles than 36 patients with community-acquired pneumonia due to *S. aureus* without PVL genes. The patients in the first group also had a more severe disease course and 75% of them died; in the other group, 47% died (*5*). Dufour et al. reported on 14 cases of community-acquired infections due to PVL-positive MRSA, suggesting the community emergence of a new superadapted *S. aureus* strain (*9*).

In our study, nine persons belonging to a population of classmates, teachers, and their family members were found to be nasal carriers of a single clone of PVL gene–positive, methicillin-susceptible *S. aureus* after six students and three of their relatives had had relapsing episodes of skin infections in 13 months. Overall, the 44% prevalence of *S. aureus* carriage in this population was higher than the expected rate of 30% (*2*) but close to it without taking the isolates belonging to the PVL-positive clone (A) into account. Although the numbers were small and this difference could be due to chance, this finding suggests an addition to the usual rate of colonization caused by the introduction of clone A into the studied population, an event that seemed to have occurred recently, as suggested by its very homogenous PFGE profile as compared with the others.

Even though the *S. aureus* responsible for infections had not been saved and could therefore not be tested, the nasal recovery of a single PVL-positive clone only from persons who had had skin infections or from their family members strongly suggests pathogenicity of this particular clone. Indeed, Prevost et al. found identical PFGE profiles in nasal and furuncle PVL-positive isolates from patients with skin infections (*10*). Although only one isolate representative of each clone was tested for PVL genes, we believe that, given the large size of the bacteriophage carrying these genes, it is very unlikely that isolates showing the same PFGE pattern could differ regarding their PVL status. In addition, the *S. aureus* found in subsequent nasal swabs from the same persons in our study belonged consistently to the same clone as the first cultured isolate, suggesting that these persons were colonized with a single clone each and that the hazard of picking one among several strains of *S. aureus* for PFGE typing was low. Similarly, Peacock et al. found that 29 of 31 patients with >1 positive nasal culture for *S. aureus* within a 1-month period harbored the same clone over time (*11*). The presence of LukD and LukE genes in addition to genes encoding for PVL was considered trivial since these toxins do not appear to be linked with specific infections (*3*).

To prevent new cases or more severe diseases, the decision was made to implement MRSA measures derived from those in use in many hospitals (*6*). These measures consisted of the repeated application of nasal mupirocin and chlorhexidine showers for those considered at risk for infection or transmission and the simultaneous use of alcohol-based hand rubs for the same persons, plus all of their classmates and teachers. Given the absence of new cases and the occurrence of only two relapses 1 year after these measures were implemented, they may have played a role in controlling this outbreak.

In summary, PVL-positive *S. aureus* may spread between persons in close contact and cause disease, mainly among otherwise healthy children or young adults. The clinical manifestations may initially correspond to skin infections and then progress to severe necrotizing pneumonia with a high death rate. Despite its limitations, this study suggests that future research should investigate whether the early identification of such strains, coupled with timely measures aimed at decolonizing carriers and interrupting person-to-person transmission, could prevent or control potentially lethal outbreaks.
